# Paederus dermatitis

**DOI:** 10.11604/pamj.2018.30.136.15189

**Published:** 2018-06-18

**Authors:** Ahmed Bouhamidi, Mohammed Boui

**Affiliations:** 1 Military Hospital of Instruction Mohammed V, Department of Dermatology, Rabat, Morocco

**Keywords:** Dermatitis, paederus, rove beetles

## Image in medicine

There were two patients, having crushed insects on their bodies, described as a two-colored big ant with three black segments (head, center and end of the abdomen) and two orange segments, which were in their accommodation. They presented with a dermatitis characterized by initial marked erythema and pruritus, with vesicles and blisters in cervical region developing after 48 hours post exposure for the first patient, while the second patient developed similar chest lesions and skin ulceration after 72 hours of exposure. Based on clinical and epidemiological features, the diagnosis of Paederus dermatitis was retained. Treatment is made with topical Trolamine, oral antihistamines, and intensive washing. The lesions improved over several days, with resolution of lesions without sequelaes 10 to 15 days later. Paederus dermatitis is a contact dermatitis that occurs due to physical contact with rove beetles of the genus Paederus. The diagnosis is based on the presence of typical clinical features combined with compatible epidemiological features. This condition is prevalent in some tropical and subtropical regions, such as Guinea-Bissau, and especially in the rainy season.

**Figure 1:**
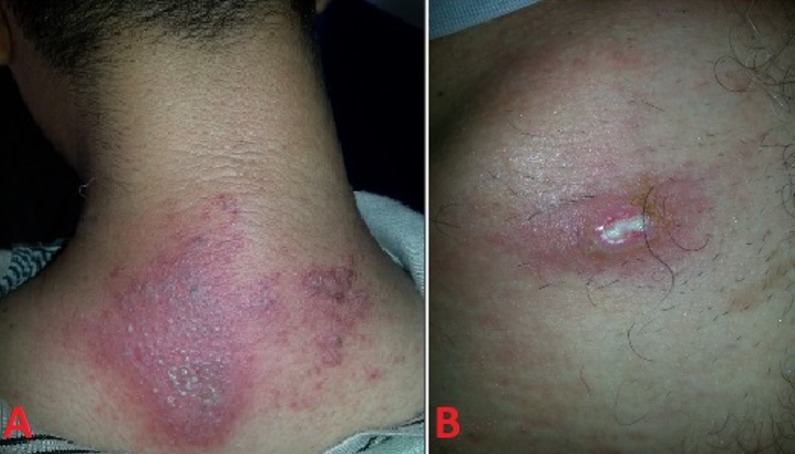
A): vesicular lesions on an erythematous base in cervical region; B): skin ulceration on an erythematous base in thoracic region

